# Randomized comparative study of suspension femoral fixation device in graft position maintenance in anterior cruciate ligament reconstruction: EndoButton CL vs TightRope RT

**DOI:** 10.1016/j.asmart.2021.05.007

**Published:** 2021-06-01

**Authors:** Yoshimasa Ono, Yusuke Sato, Hiroki Mukai, Takahiro Enomoto, Seiji Kimura, Ryosuke Nakagawa, Ryuichiro Akagi, Yosuke Inaba, Yohei Kawasaki, Seiji Ohtori, Takahisa Sasho

**Affiliations:** a Department of Orthopaedic Surgery, Graduate School of Medicine, Chiba University, Japan; b Department of Diagnostic Radiology and Radiation Oncology, Chiba University, Japan; c Biostatistics Section, Clinical Research Center, Chiba University, Japan; d Musculoskeletal Disease and Pain, Preventive Medical Sciences, Chiba University, Japan

**Keywords:** Anterior cruciate ligament reconstruction, Cortical suspensory fixation device, Fixed loop, Adjustable loop

## Abstract

**Background:**

In double-bundle anterior cruciate ligament reconstruction (ACLR), fixed-loop and adjustable-loop cortical suspensory devices are commonly used to fix the soft graft on the femoral side. However, few studies have compared in vivo elongation of the two devices. The purpose of this study was to determine whether EndoButton CL (EB) and TightRope RT (TR), the suspensory fixation devices used in ACLR, maintained their length in vivo from the time of surgery through the postoperative period in a randomized controlled trial.

**Methods:**

This study prospectively incorporated 30 patients undergoing initial ACLR at a single center. Participants were divided into two groups using a stratified randomization method with age and sex as assignment adjustment factors. EB or TR was used for fixation of the soft graft on the femoral side. The primary endpoint was to compare the elongation distance of the suspensory device. MRIs were taken within seven days after ACLR and 3,6,12 months postoperatively and measured by a radiologist in a blinded fashion. Secondary endpoints included the side-to-side difference in anterior translation, one leg hop test (HOP index), Lachman test, lateral pivot shift test, and Lysholm score one year postoperatively.

**Results:**

Twenty-eight patients (EB, n = 13; TR, n = 15) were followed for one year. There was no significant difference between EB and TR groups in elongation from the immediate postoperative period to 3, 6, 12 months after surgery. However, the non-inferiority of TR to EB (non-inferiority margin: 1.5 mm) was not proved by the difference in measured elongation between the two groups (TR – EB, lower 95% CI. AM: 1.80 mm; PL: 1.86 mm) at 6 months. There was no significant difference in anterior translation, HOP index, Lachman test, lateral pivot shift test, or Lysholm score.

**Conclusion:**

EB and TR had similar graft retaining ability in vivo for 12 months, but the non-inferiority of TR against EB was not verified statistically.

## Introduction

Use of suspensory devices are popular in anterior cruciate ligament reconstructions (ACLR) when fixing soft graft material to the femoral side.^1^^,^^16^ The devices consist of the combination of a small rectangular metal button that firmly contacts the cortex of the femur and a thread that connects the button and the graft. EndButton CL™ (EB, Smith & Nephew, Andover MA, USA) and Tightrope RT™ (TR, Arthrex, Naples FL, USA) are the two major devices used. The former maintains the distance between the button and the graft with a prefabricated fixed loop thread and the latter with an adjustable loop that constricts in one direction by an intrinsic “Chinese trap” mechanism. Contrary to their theoretical stability, previous biomechanical studies have shown elongation of both of the devices^7^ after cyclic biomechanical tests. Smaller elongation is desirable to minimize failure of the ACLR due to graft insufficiency. Cyclic displacement tests to evaluate the elongation have been conducted using the device alone or the device loaded with bovine or porcine tendons. Most isolated device studies have reported favorable outcomes for EB and most studies have reported no statistically significant difference between EB and TR devices loaded with tendon.^2^^,^^3^^,^^5^, ^6^, ^7^, ^8^^,^^13^^,^^15^ All these studies provide important insights for performing ACLR.

To address what is happening in the ACLR knee joints with these devices in vivo, we conducted a prospective study in which graft displacement after double bundle ACLR with the two types of cortical suspension devices was compared by examining graft position with the use of three-dimensional MRI. Patients were allocated to the use of EB or TR randomly. The distance between the femoral cortex and the tip of the graft was measured at four time points (within one week, three, six and twelve months after ACLR).

## Methods

This randomized controlled study was performed at our institute between March 2016 and December 2018. The study included patients who required surgical intervention for unstable knees due to ACL injuries. Patients were eligible if they met all the following inclusion criteria: (1) less than 50 years of age with definite closure of epiphyseal lines, (2) grade 2 or 3, or no endpoint on the Lachman test, (3) a positive lateral pivot shift test, (4) a torn ACL on MRI examination, (5) more than 120° of knee joint range of motion. The exclusion criteria were: (1) those who chose conservative treatment, (2) those who preferred to receive surgeries with graft materials other than the hamstrings tendon, (3) those who were instructed to restrict activities due to medical conditions, (4) re-injury cases, and (5) those with a previous history of ipsilateral ACL injury.

After baseline measurements, participants were allocated to either the group with fixed-loop button fixation (EB) or with adjustable-loop button fixation (TR) as a femoral-side fixation device. Stratified randomization was used for assignment to the treatment group. This study was approved by our institutional review board, and all the patients provided written informed consent.

### Surgical technique

All ACL reconstructions were performed by one of six co-authors. Patients underwent arthroscopic anatomical double-bundle reconstruction using hamstring tendons. After conventional arthroscopic examination through the anteromedial and anterolateral portals, the hamstring tendon was harvested, and two double-bundle grafts were prepared. The femoral bone tunnels were created by the outside-in technique. Using a femoral aimer (Arthrex), two guide wires were inserted from the outside of the lateral cortex of the femur to footprints of the anteromedial (AM) and posterolateral (PL) bundles of the ACL. After confirming that the two tips of the wires were placed in the desired positions, two bone tunnels were drilled over the guidewires from the outside of the lateral femoral cortex to allow passage of a retrograde drill (FlipCutter, Arthrex) to create sockets 15 mm long. The opening of the tibial AM tunnel was created just posterior to the anterior margin of the ACL remnant, and that of the PL tunnel was created approximately 12–15 mm anterior to the anterior border of the PCL while referring to the attachment of the anterior horn of the lateral meniscus. After creating two femoral and two tibial tunnels, each graft was passed through each tunnel. Either EB or TR was used for the femoral fixation device and TR was used as the tibial fixation device in all the cases according to previously reported graft configuration.^18^ The initial graft tension was applied manually while arthroscopically verifying the tension of the graft. After the initial fixation of the graft, the knees were passively extended and flexed from 0 to 90° ten times to reveal loss of initial tension that would occur with the beginning of early-phase rehabilitation. The tension of the graft was re-checked arthroscopically and tightened again with the tibial-side TR until reaching the initial tension.

### Rehabilitation protocol

The postoperative rehabilitation protocol was the same for all patients. Range of motion training of the knee joint and strength training of the quadriceps muscles started on the 2nd day after the surgery, and half weight-bearing walking started on the 3rd day. Full weight-bearing began two weeks postoperatively. Three months after the surgery, patients were allowed to start light exercise such as jogging without rotational movement. Four months after surgery, full speed running in a straight line was allowed. Six months after surgery, patients were allowed to start activities related to pre-injury sports, such as jumping and turning. Patients were permitted to start participating in sports activities eight months after surgery if muscle strength had sufficiently recovered. Full return to sports was usually at ten months.

### Primary outcomes

**Fig. 1:**
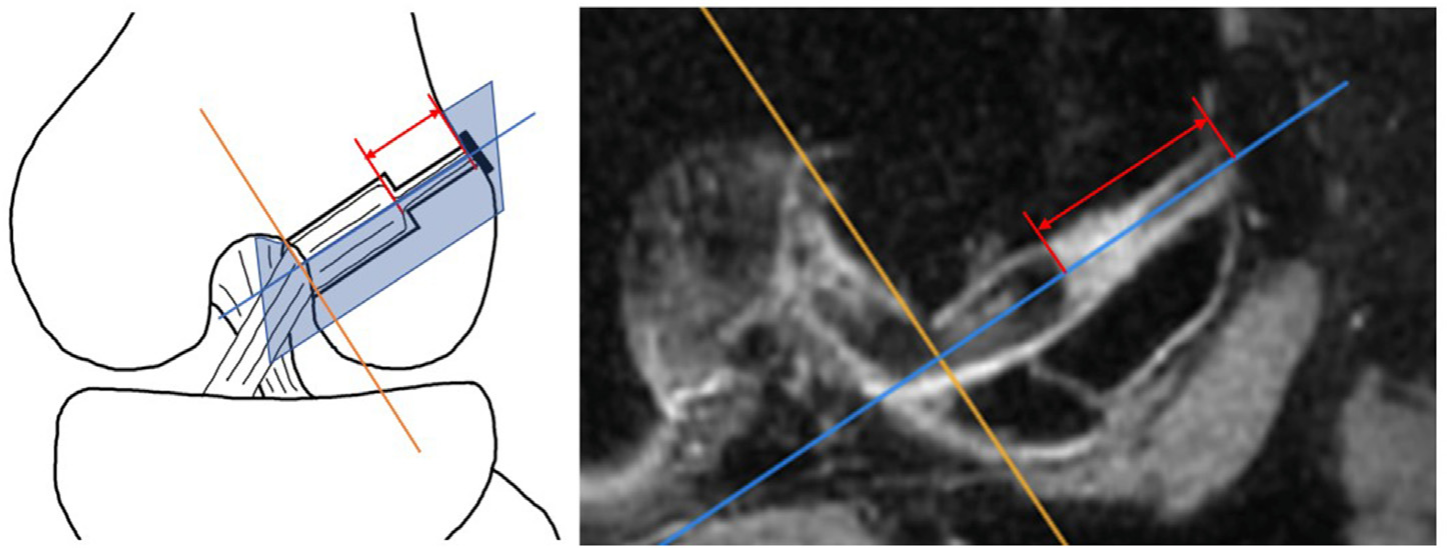
Schematic and MRI measurements of the distance from the reconstructed ligament to the lateral cortical bone of the femur. Red line: Distance between the lateral cortical surface of the femur and the tip of graft; Blue line: Axis of the femoral tunnel; Yellow line: Axis perpendicular to femoral tunnel axis. (For interpretation of the references to colour in this figure legend, the reader is referred to the Web version of this article.)

Subjects received three-dimensional isotropic, proton emphasis fat suppression MR images (132 slices) captured by a 3.0T machine (Discovery MR750, GE Healthcare, USA) within seven days, three, six, and twelve months after surgery. Advantages of three-dimensional isotropic imaging include improved through-plane spatial resolution and the ability to generate high-quality reformats to yield multiplanar images from the original imaging dataset. Graft position was assessed by reconstructing axial, sagittal, and coronal sections, respectively, using imaging software (Aquarius NET Viewer, TeraRecon, USA). Then the desirable slices that ran through the axis of the femoral tunnel were reconstructed and the distance between the lateral cortical surface of the femur and the tip of graft was measured (Fig. 1). An experienced musculoskeletal radiologist made the measurements.

Elongation was assessed as the difference in the distance measured immediately after surgery and at three, six, and twelve months postoperatively. We considered six months is the most important endpoint because activities of the participants were well under control within this period and bone tunnel healing was presumably completed or nearly completed by this time. Thus, non-inferiority was assessed at six months.

### Secondary outcomes

Patients underwent pre- and postoperative objective and subjective evaluations. Objective evaluations were made by measuring the side to side difference in anterior translation of the tibia measured with KSM-100 (Sigmax, Japan), the HOP index,^4^ the Lachman test, and the lateral pivot shift test.

More specifically, the KSM-100 measurements at a maximum manual pull of 15° of flexion were performed before surgery, at three, six, and twelve months after surgery, and recorded as the difference between the injured and uninjured legs. The hop test was performed at six and twelve months, with the average of three standing long jumps with one leg on both injured and uninjured sides used as the HOP distance. The ratio of the injured side to the uninjured side was used as the HOP index.

The Lysholm score^11^ was used as a subjective evaluation. Patients' satisfaction and sports performance levels were measured in one mm increments on a 100 mm visual analog scale one year after surgery and compared to preoperative levels.

Postoperative complications that might need additional surgeries were recorded, such as severe loss of knee motion, tear of the reconstructed ACL, and ACL injures of the contralateral knee.

### Sample size

The necessary sample size was calculated before starting the study. Although there had been no report on the elongation of suspensory fixation devices in the long-term in the human knee joint in vivo, short-term mechanical tests with the suspension fixation devices reported elongation of 1.05 ± 0.05 mm (mean ± standard deviation) and 2.2 ± 0.6 mm in the EB and TR groups, respectively.^8^ Because the load in the human knee joint was considered to be smaller than the load used in the mechanical tests, the estimated changes in the EB group and the TR group were set to be 0.5 mm and 1.1 mm, respectively, with standard deviations of 0.6. Therefore, the margin of non-inferiority was determined to be −1.5 mm. This value would be within an acceptable range from the clinical standpoint. The clinical outcome was considered good if the postoperative anterior instability was within two mm compared with the healthy side, and was considered excellent if within one mm.^12^ Precise association of the elongation with anterior translation had not been determined but we assumed the amount of anterior translation would be less than the amount of elongation that occurred in the bone tunnel. The calculated sample size based on these considerations was 13 cases per group (n = 26 total cases) with a one-tailed significance level of 0.025 and power of 0.8 in the two-sample *t*-test. Assuming a dropout rate of 15%, 15 cases per group was set as the target sample size.

### Statistical analysis

Continuous variables were summarized as the mean and SD and categorical variables as frequencies and proportions. The main purpose of the analysis in this study was to show that the TR group was not inferior for elongation distance (non-inferiority) to the EB group. The elongation of each AM and PL bundles was analyzed using an unpaired *t*-test and the two-sided 95% confidence interval of the difference in elongation (TR group - EB group) was calculated. If the lower limit of the calculated confidence interval was greater than −1.5 mm, which was the non-inferiority margin, the hypothesis that TR was not inferior to EB was rejected. Secondary outcomes such as KSM-100 measurements, HOP index, Lachman test, lateral pivot shift test, and Lysholm score were analyzed for the purpose of complementing the main analysis of this study. KSM-100, HOP index, and Lysholm score were analyzed using the unpaired *t*-test. The Lachman test and lateral pivot shift test were analyzed using Fisher's exact test. Safety was examined using the safety analysis set, which was defined as any patient who had at least one safety assessment at after baseline measurements. All data were analyzed using SAS (64 bit SAS 9.4 for Windows) and a two-sided p-value of <0.05 was considered statistically significant.

## Results

**Fig. 2:**
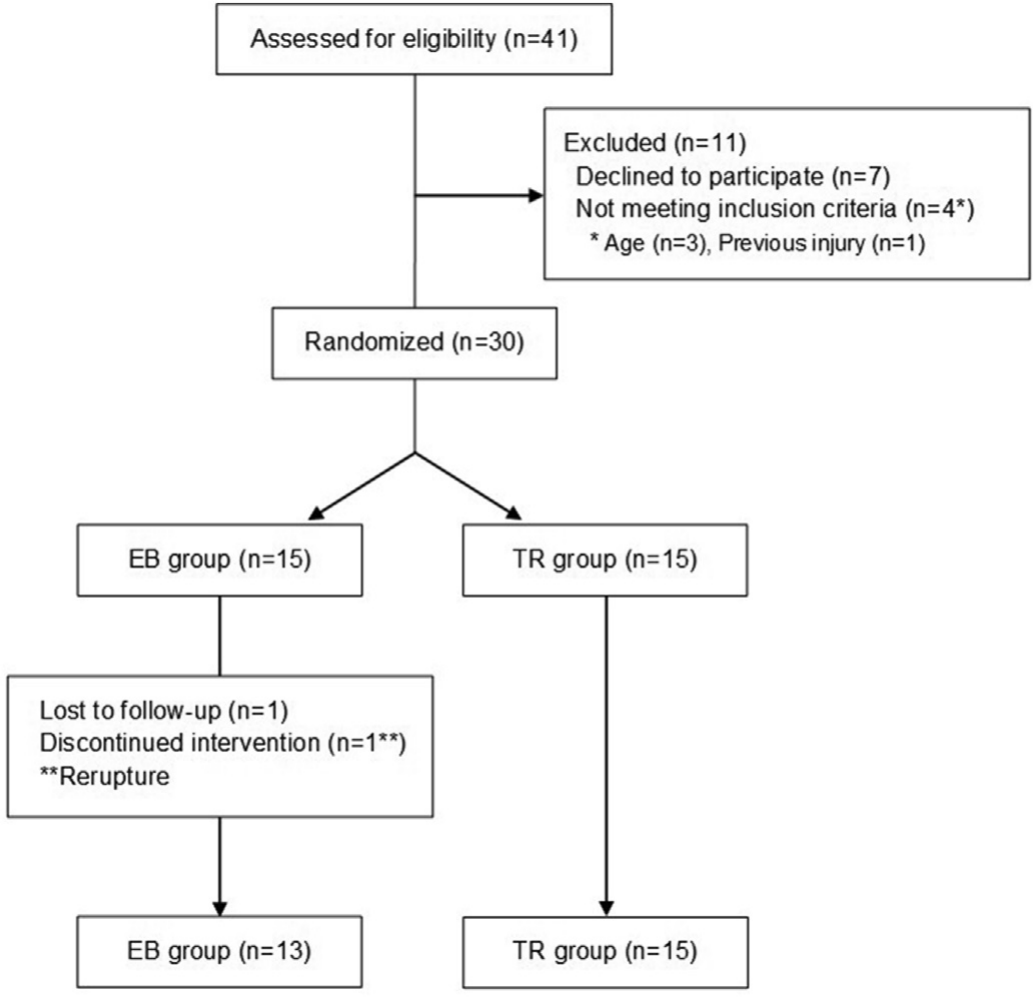
CONSORT flow diagram. EB, EndoButton; TR, TightRope. (This figure is uploaded separately.)

A total of 41 patients were referred to our institute with ACL injuries. Three patients were excluded because they were over 50 years old or skeletally immature. One patient was excluded due to a previous injury to the ipsilateral knee. Seven patients refused to participate in the study. This left 30 patients included in the study (15/group). Two of the 30 patients were not included in the evaluation. One in the EB group re-ruptured within 8 months and another in this group was lost to follow-up (Fig. 2). There were no significant differences in the demographic data in relation to age, sex, the Lachman test, the lateral pivot shift test, or the Lysholm score. The EB group was affected significantly more often on the left side (Table 1).

**Table 1:** Patient characteristics and preoperative evaluation.

Preoperative characteristics	EndoButton CL	TightRope RT	*P*
Age (years)	25.2 ± 9.6^a^	25.7 ± 8.4^a^	0.88
Sex (male/female)	6/7	8/7	0.70
Side (right/left)	3/10	10/5	0.03
Lachman test grade (1/2/3)	2/10/1	1/12/2	0.83
Lateral pivot shift test (+/-)	12/1	15/0	0.46
Lysholm score	69.7 ± 22.4^a^	71.6 ± 19.3^a^	0.70

a Mean ± standard deviation.

### Primary outcome

The difference of measured elongation between the TR group and the EB group was −1.8 mm for AM and −1.86 mm for PL at the lower end of the mean 95% confidence interval. The non-inferiority on elongation of the TR group against the EB group was not proven because the non-inferiority margin was set at −1.5 mm. Although the non-inferiority of TR to EB could not be proven, there was no significant difference between the two groups at three, six, and twelve months postoperatively (Table 2).

**Table 2:** Elongation from immediately after surgery to three, six and twelve months after surgery.

Time elapsed and bundles		EB group (mm)	TR group (mm)	TR – EB (mm)	*P*
3 months					
	AM bundle (95% CI)	−0.04 (−0.93, 0.85)	−0.29 (−0.87, 0.30)	−0.24 (−1.26, 0.77)	0.61
	PL bundle (95% CI)	0.43 (−0.50, 1.37)	−0.23 (−0.91, 0.46)	−0.66 (−1.73, 0.41)	0.21
6 months					
	AM bundle (95% CI)	0.53 (−0.48, 1.53)	−0.002 (−0.89, 0.85)	−0.54 (−1.80, 0.71)	0.19
	PL bundle (95% CI)	0.80 (0.003, 1.60)	0.12 (−0.84, 1.07)	−0.68 (−1.86, 0.49)	0.23
12 months					
	AM bundle (95% CI)	0.85 (−0.48, 2.19)	1.05 (0.07, 2.03)	0.20 (−1.33, 1.72)	0.26
	PL bundle (95% CI)	0.66 (−0.14, 1.46)	0.41 (−0.23, 1.04)	−0.25 (−1.21, 0.71)	0.59

AM, anteromedial; PL, posterolateral; CI, confidence interval.

### Secondary outcome

There were no significant differences in the HOP index, KSM-100, the Lachman test, the lateral pivot shift test, or the Lysholm score (Table 3).

**Table 3:** Clinical evaluation one year after surgery.

Items examined	EndoButton CL	TightRope RT	*P*
HOP index	0.90 ± 0.13^a^	0.83 ± 0.19^a^	0.27
KSM-100 (mm)	0.5 ± 1.7^a^	1.0 ± 2.5^a^	0.51
Lachman test grade (1/2)	12/1	12/3	0.60
Lateral pivot shift test (+/-)	13/0	14/1	1.00
Lysholm score	94.9 ± 8.3^a^	86.7 ± 13.3^a^	0.07

a Mean ± standard deviation.

## Discussion

This in vivo study revealed that the distance between the femoral cortex and graft apex did not differ between the uses of EB or TR from three to twelve months after double-bundle ACL reconstruction, but the non-inferiority of TR against EB was not verified statistically at six months.

Equivalent clinical results have been reported with the use of a fixed-loop device vs an adjustable loop device as a suspensory femoral side fixation for a soft graft in ACLR. In 2018, Ranjan et al. reported equivalent International Knee Documentation Committee, Lysholm scores, anterior translation, and side-to-side differences in their two year follow up study comparing EB and TR single bundle reconstruction.^16^ Ahn et al. reported comparable graft healing assessed by MRI signal intensity at follow up as well as clinical results using RetroButton as a fixed-loop device.^1^ The present study also found no differences in clinical outcomes between the two groups.

Biomechanical studies with isolated devices suggested a smaller displacement with EB. In 2018, Houck reported in their review paper that all five studies reported favorable displacements for EB in device only experiments.^7^ Retaining the initial graft position with suspensory devices is a key factor for successful ACLR, but reasons for the discrepancy between clinical studies and device only mechanical studies have not been determined. The distance between the femoral cortex and graft apex assessed in the present study was mostly attributable to the device itself, although the femoral cortex and the thickness of the doubled over graft apex were other possible factors that could affect the measurement. Interestingly, biomechanical studies that dealt with isolated devices as well as graft construct under simulated ACL reconstruction situations using porcine knees and bovine tendons resulted in similar conclusions that EB was superior in device only analyses but elongation between EB and TR was not different when studied only as construct models.^3^^,^^5^ Reasons for this discrepancy were not sought in these studies but when the same loads were applied to the devices directly and to the distal end of the graft, actual loads on the devices were lower in the latter situation. We can only assume that the existence of the bone tunnel and/or soft tissue graft had a buffering effect. Considering that graft constructs were pulled parallel to the bone tunnel in these studies, the existence of a soft graft seemed to have a much greater buffering effect. The two to three-fold higher stiffness of the device itself compared to the construct might account for this.^6^^,^^15^ In vivo, reconstructed grafts are forced to bend at the exit of the femoral bone tunnel at various angles depending on the activity. It may not be possible to replicate the same situations *ex vivo*.

The actual elongation for both EB and TR was less than one mm for both AM and PL, similar to the cyclic elongation values reported by Gotschi et al.^6^ *Ex vivo* biomechanical studies using only the construct model measured initial elongation and cyclic elongation separately, and the initial elongation was larger than the cyclic elongation.^5^^,^^6^^,^^13^ This was not the case in the present study. We hypothesized that this was mostly due to surgical technique in which grafts were re-tensioned when any laxity was observed arthroscopically after 10 passive flexion-extension knee motions at the final step of the reconstruction.^18^ This lessened the initial elongation.

The present study assessed elongation up to twelve months when bone-to-tendon healing presumably had completed. We presumed not much change would occur after this point and did not examine thereafter. Animal studies have shown that 8–12 weeks is required for healing of soft graft in a bone tunnel,^19^^,^^20^ but there is not much evidence of this from human studies. In 2015, Lazarides et al. reported histological examination of tendon-bone-healing using a whole knee specimen retrieved from a 14-year-old boy who was diagnosed with osteosarcoma of the proximal tibia four months after ACLR with a soft tissue graft and EB.^10^ They found early integration of the graft on the tibial side but little evidence of healing on the femoral side after four months. Although neoadjuvant chemotherapy might negatively affect the healing process, this may suggest a difference in healing times between animal models and humans. Other than this single case report, histological specimens from human samples have been restricted to retrieved specimens from failure cases. In 2000, Petersen et al. reported histological examination of femoral and tibial bone tunnels from 14 cases following revision ACLR.^14^ Six were reconstructed with hamstring graft material with the use of EB on the femoral side 6–33 months after the initial surgeries. They found fibrous insertion of the grafts indicative of completion of healing regardless of time from surgery, suggesting that bone to tendon healing occurs as early as six months postoperatively. In 2003, Robert et al. reported histological examination of 12 revision cases. They found formation of a fibrovascular interface at three months and formation of a fibrous interface with mature indirect anchorage after ten months.^17^ MRI was used to assess longitudinal changes of the bone-to-tendon junction or graft maturation non-invasively in clinically successful cases. Kanamura conducted MR angiography on 100 cases of ACLR with hamstring grafts and reported formation of new blood vessels connecting the bone tunnel to the tendon graft, indicative of fibro-vascular interface, by three months postoperatively.^9^ Thus, bone-to-tendon healing in humans probably completes between three and ten months. Six months we considered to be the most important time point might be too early to assess healing but still was within the range of times required for complete healing found in other studies. Twelve months seemed to be enough for this type of study.

This study had several limitations. First, baseline acquisition of MRI was performed within seven days after ACLR. Ideally MRI immediately after surgery would be preferable but that was difficult in this human study. Second, measurement of distance was performed on MR images and reconstructed scans along the tunnel which creates inherent errors in the measurement process. We attributed measured elongation value solely to suspensory device but it might include multiple factors. Third, the reference point of the measurements was the surface of the femoral cortex and apex of the doubled over graft. Thus, the measured distance was not elongation of the device but an approximation. Forth, we set −1.5 mm as non-inferiority margin which lacked solid reference due to the paucity of previous studies.

## Conclusion

EB and TR had similar graft retaining ability in vivo for twelve months but non-inferiority of TR against EB was not verified statistically at six months.

## Declaration of competing of interest

This study was financially supported by Arthrex Japan LLC as an investigator initiated research grant (ID: APAC6).
